# Complete genome sequences of *Campylobacter hepaticus* strains USA1 and USA5 isolated from a commercial layer flock in the United States

**DOI:** 10.1128/mra.00919-24

**Published:** 2025-04-17

**Authors:** Chaitanya Gottapu, Orhan Sahin, Lekshmi K. Edison, Mariela Elizabeth Srednik, Subhashinie Kariyawasam

**Affiliations:** 1 Comparative, Diagnostic, and Population Medicine, College of Veterinary Medicine, University of Florida, Gainesville, Florida, USA; 2 Department of Veterinary Diagnostic and Production Animal Medicine, Iowa State University, Ames, Iowa, USA; 3 Department of Veterinary Microbiology and Preventive Medicine, Iowa State University, Ames, Iowa, USA; University of Maryland School of Medicine, Baltimore, Maryland, USA

**Keywords:** genomes, *Campylobacter hepaticus*, Illumina, nanopore technology

## Abstract

*Campylobacter hepaticus* is known to cause spotty liver disease (SLD) in layer chickens around peak production. Here, we report the complete genome sequences of two *C. hepaticus* strains, USA1 and USA5, from the United States. The genomes comprise chromosomes of 1,508,744 and 1,531,174 bp, respectively, with a G + C content of 28%.

## ANNOUNCEMENT

Spotty liver disease (SLD) is an acute infectious disease of layer chickens, which is caused by *Campylobacter hepaticus* (1, 2). While bacterial genome sequencing has advanced our understanding of disease pathogenesis and therapeutic targets, only a few *C. hepaticus* genomes are publicly available (3–5). This study reports the complete genomes of two *C. hepaticus* strains, USA1 and USA5, to support future research.

*C. hepaticus* strains USA1 and USA5 were isolated in 2018 from the bile of 57- and 27-week-old commercial layer chickens with SLD, originating from Texas and an undisclosed location in the United States, respectively, using 5% sheep blood agar (Remel, Kansas) at 37°C under microaerophilic conditions for 3 days, at the Veterinary Diagnostic Laboratory at Iowa State University. The isolates were confirmed as *C. hepaticus* by a PCR targeting the glycerol kinase gene (*glk*) and 16S rRNA gene sequencing (1). Genomic DNA was extracted from bacteria grown under the initial culture conditions using Quick-DNA HMW MagBead Kit (Zymo Research, California). DNA purity was verified with a NanoDrop (Thermo Fisher Scientific, Waltham, MA), and library preparation and sequencing were conducted at SeqCenter (Pittsburgh, PA, USA) using Oxford Nanopore (Oxford, UK) and Illumina (San Diego, CA, USA) technologies. Nanopore libraries were prepared using the PCR-free Oxford Nanopore Ligation Sequencing Kit (SQK-NBD114.24) in conjunction with the NEBNext Companion Module (New England Biolabs, Ipswich, MA, USA), and sequencing was performed on an Oxford Nanopore MinION Mk1B sequencer using an R10.4.1 flow cell (6). Illumina libraries were prepared using tagmentation and PCR-based Illumina DNA Prep kits with custom IDT (Integrated DNA Technology, Coralville, IA, USA) 10 bp unique dual indices (UDIs) targeting an insert size of 280 bp. Sequencing was performed on the Illumina NovaSeq X Plus sequencer in multiplexed shared-flow-cell runs, generating 2 × 151 bp paired-end reads. Nanopore libraries were prepared without DNA fragmentation or size selection. Nanopore reads were quality-controlled with Porechop v0.2.4 (7) for adapter trimming and assembled using Flye2 v2.9.2 under the nano-hq model (8). Guppy v6.5.7 was used for base-calling (9). The genome assemblies for USA1 and USA5 achieved ONT mean read length values of 1,1120.183 and 8,692.119 and N50 values of 1,508,744 and 1,531,174 bp, respectively. Polishing with Illumina reads via Pilon (10) produced final contigs of 1,508,744 and 1,531,174 bp. Annotation was completed using the NCBI Prokaryotic Genome Annotation Pipeline (PGAP-6.7) (11). For USA1, the reads included 3,393,557 (Illumina) and 146,860 (ONT), while for USA5, there were 2,509,316 (Illumina) and 294,008 (ONT) reads. The assemblies were circularized using Circulator v1.5.5 (12), identifying *dnaA* start sites and overlaps. All tools used default parameters except for Flye2, which was set with an asm-coverage 50 and a genome size of 6,000,000. The final assembly produced 1,508,744 and 1,531,174 bp contigs for USA1 and USA5 strains, respectively.

Analysis revealed that *C. hepaticus* strain USA1 has 1,513 ORFs (1,458 CDS, 9 rRNAs, 43 tRNAs, and 3 noncoding RNAs), while USA5 has 1,549 ORFs (1,494 CDS, 9 rRNAs, 43 tRNAs, and 3 noncoding RNAs). Both chromosomes exhibit an average G + C content of 28.0%.

## Data Availability

The complete annotated chromosomes of USA1 and USA5 have been deposited in the NCBI GenBank under the accession numbers CP166729 and CP166688, BioProject accession number PRJNA1145931, and BioSample accession numbers SAMN43092218 and SAMN43080279, respectively. Raw sequences have been deposited at the NCBI Sequencing Read Archive under accession numbers SRX26289172 (Nanopore) and SRX26289170 (Illumina) for USA1 and SRX26289173 (Nanopore) and SRX26289171 (Illumina) for USA5, respectively. The 16S rRNA sequences were deposited with accession numbers PQ569979 and PQ569980 for USA1 and USA5, respectively.
